# Characterization and functional analysis of two novel 3-hydroxy-3-methylglutaryl-coenzyme A reductase genes (*GbHMGR2* and *GbHMGR3*) from *Ginkgo biloba*

**DOI:** 10.1038/s41598-019-50629-8

**Published:** 2019-10-01

**Authors:** Shen Rao, Xiangxiang Meng, Yongling Liao, Tian Yu, Jie Cao, Junping Tan, Feng Xu, Shuiyuan Cheng

**Affiliations:** 1 College of Horticulture and Gardening, Yangtze University, Jingzhou, 434025 China; 2 National R&D for Se-rich Agricultural Products Processing Technology, Wuhan Polytechnic University, Wuhan, 430023 China; 3 Serun Health Industry Group, Enshi, 445000 China

**Keywords:** Plant molecular biology, Plant biotechnology

## Abstract

Terpene trilactones (TTLs) are the main secondary metabolites of *Ginkgo biloba*. As one of the rate-limiting enzymes in the mevalonic acid (MVA) pathway of TTL biosynthesis, 3-hydroxy-3-methylglutaryl-coenzyme A reductase (HMGR) catalyzes the 3-hydroxy-3-methylglutaryl coenzyme A to form MVA. In this study, two cDNA sequences of *HMGR* genes, namely, *GbHMGR2* and *GbHMGR3*, were cloned from *G. biloba*. The protein sequences of GbHMGR2 and GbHMGR3, which contain several functional domains, were analyzed. Regulatory elements related to light, hormone, and stress response were detected in the promoter regions of *GbHMGR2* and *GbHMGR3*. The catalytic activity of these genes was verified by a functional complement experiment in yeast. Quantitative real-time PCR (qRT-PCR) showed the distinct expression patterns of the two genes in different organs. The TTL contents in the organs were detected by high-performance liquid chromatography– evaporative light scattering detector. *GbHMGR2* and *GbHMGR3* were responded to cold, dark, methyl jasmonate (MJ), abscisic acid (ABA), salicylic acid (SA), and ethephon (Eth) treatments. The TTL contents were also regulated by cold, dark, MJ, ABA, SA, and Eth treatment. In conclusion, *GbHMGR2* and *GbHMGR3* may participate in the MVA pathway of TTL biosynthesis.

## Introduction

*Ginkgo biloba* is an important medicinal plant, and its leaf extracts are widely used in clinical medicine. The most popular *G. biloba* extract product is EGB761, which contains 24% ginkgo flavone glucosides and 6% terpene trilactones (TTLs) and exhibits protective properties against neuronal and vascular damages^1^. TTLs, including ginkgolide and bilobalide, are the main secondary metabolites of *G. biloba* and important active ingredients of EGB761. In addition, TTLs are natural antagonists of platelet-activating factor and are the preferred natural drug for the treatment of cardiovascular diseases^2,3^. However, the TTL content in *G. biloba* organs is extremely low^4^, and TTLs are difficult to synthesize chemically because of their complex chemical structures. Hence, methods that improve TTL content are the focal point in studies on *G. biloba*.

**Figure 1:**
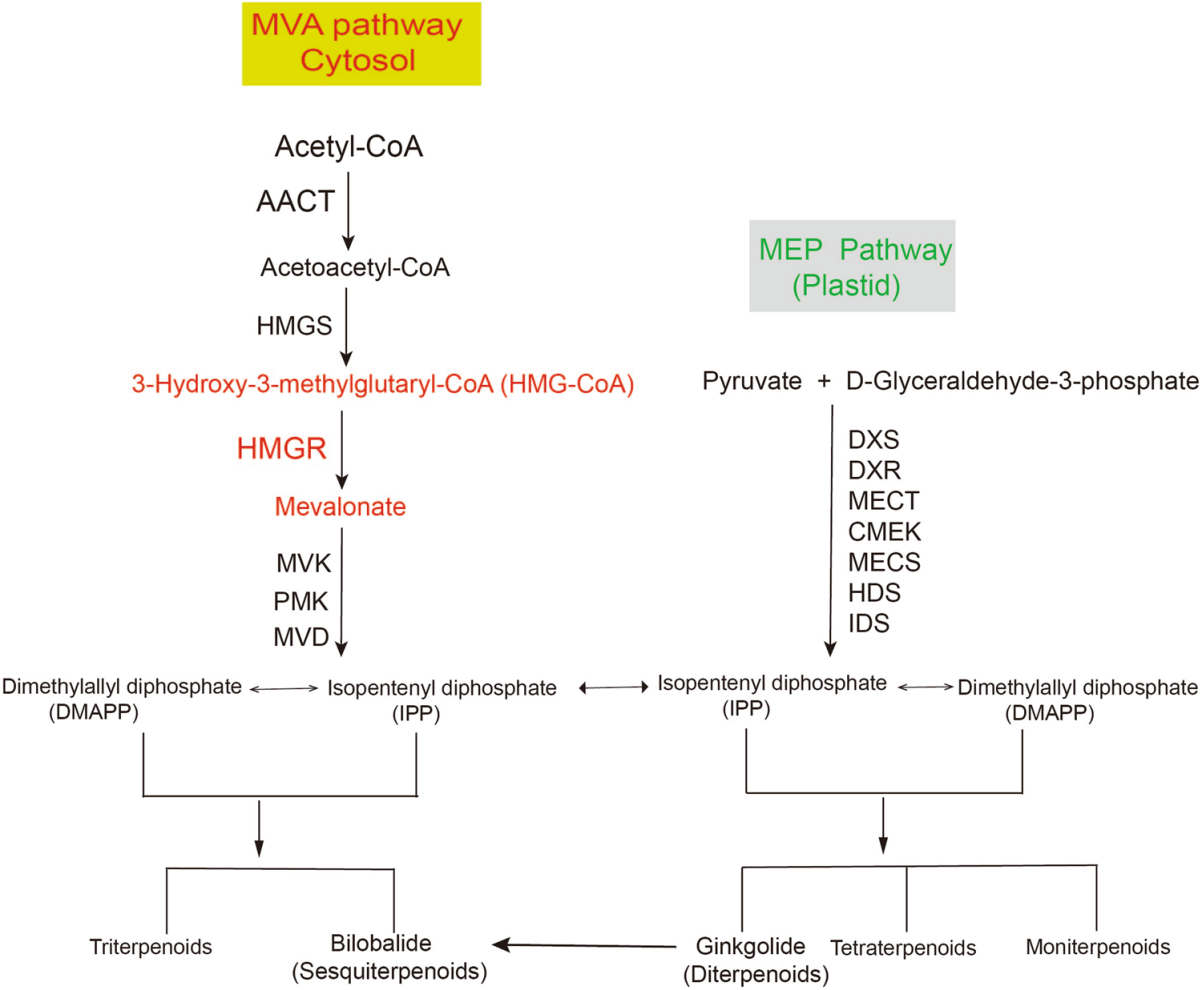
Metabolic pathways of terpenoids.

Terpenoids are generally synthesized through the mevalonic acid (MVA) and methylerythritol phosphate (MEP) pathways in plants. Monoterpenes, diterpenes, and tetraterpenes are generated through the MEP pathway, whereas sesquiterpenes and triterpene are produced through the MVA pathway^5^. Terpenoid content can be effectively increased by overexpressing a key gene in the terpenoid synthesis pathway or regulating the expression of multiple terpene synthase genes by transcription factors^6,7^. 3-Hydroxy-3-methylglutaryl-coenzyme A reductase (HMGR) is one of the rate-limiting enzymes in the MVA pathway of terpenoid biosynthesis and catalyzes 3-hydroxy-3-methylglutaryl coenzyme A (HMG-CoA) to form MVA^8^ (Fig. 1). Overexpression of the *HMGR* gene can effectively increase the terpenoid content in plants. For example, the HMGR activity and sterol content of tobacco plants transgenic with rubber *HMGR* were increased by four to eight and six times, respectively^9^ (Schaller *et al*. 1995). Overexpressing *Catharanthus roseus HMGR* in *Artemisia annua* increased the artemisinin content by 38.9%^10^. *Arabidopsis thaliana HMGR* overexpression in *Lavandula latifolia* increased the essential oil and sterol contents^11^. Therefore, the overexpression of the *HMGR* gene shows potential in improving the TTL content of *G. biloba*.

In 1989, Caelles *et al*.^12^ first isolated the *HMGR* gene from *Arabidopsis*. To date, the *HMGR* genes have been isolated from more than 60 plants, including angiosperms and gymnosperms^4,13^. The plant *HMGR* genes generally appear under *HMGR* gene family, and *HMGR* homologous genes of the same species are differentially expressed in various tissues under diverse external stimulations^14^. For example, methyl jasmonate (MJ) depresses the expression of *Camptotheca acuminate HMGR1* (*CaHMRG1)* but not those of *CaHMGR2* and *CaHMGR3*^15^. Under normal conditions, the expression of *CaHMRG1* is detected in the reproductive organs and leaves, but *CaHMRG2* expression is found in all tissues^16^. Hence, a potential *HMGR* gene together with genetic engineering biotechnology can be used to increase terpenoid content by investigating the members of *HMGR* family and understanding the biological functions of each member and their relationship in *G. biloba*. In 2006, Shen *et al*.^17^isolated and characterized *HMGR* from *G. biloba* for the first time, they discussed that *GbHMGR* was lowly expressed in a tissue-specific manner, because this gene was found only in root. We have also previously cloned and characterized *GbHMGR1* and demonstrated that this gene is significantly related to the TTL content in *G. biloba*^4^. However, whether other HMGR genes exist in *G. biloba*, and whether such genes are related to the synthesis of TTLs remains unclear. Thus, in this study, two novel cDNAs of *HMGR* (*GbHMGR2* and *GbHMGR3*) were cloned and characterized from *G. biloba*. Yeast functional complementation of *GbHMGR2* and *GbHMGR3* was conducted to identify the functions of their proteins. Quantitative real-time PCR (qRT-PCR) was applied to determine the expression profiles of *GbHMGR2* and *GbHMGR3* in different tissues under various treatments, namely, cold, dark, MJ, salicylic acid (SA), ethephon (Eth), and abscisic acid (ABA) treatments. The TTL contents in the tissues and leaves under stress and hormone treatments were also determined. The results of this study will help in elucidating the functions of *GbHMGR2* and *GbHMGR3* and provide further insights into the biosynthesis of TTLs.

## Results

### Characterization and phylogenetic analysis of GbHMGR2 and GbHMGR3 proteins

*GbHMGR2* measures a full length of 2260 bp and contained a 1746 bp open reading frame (ORF) encoding a 581-amino-acid protein, whereas *GbHMGR3* features a full length of 2228 bp with a 1719 bp ORF encoding a 572-amino-acid protein. The sequences are similar to those of the unigenes filtered from the *G. biloba* transcriptome database (GeneBank accession number SRX1982952). BLASTn search revealed that these genes are highly homologous with the *HMGR* genes of other plants. Thus, these cloned genes are members of *HMGR* family and therefore were designated as *GbHMGR2* (GeneBank accession number MH091702) and *GbHMGR3* (GeneBank accession number MH091703).

The predicted GbHMGR2 and GbHMGR3 proteins comprise of 581 and 572 amino acids, respectively. The theoretical isoelectric point and molecular weight of the predicted GbHMGR2 protein were 8.18 and 61.7 KDa, respectively, whereas those of GbHMGR3 were 6.92 and 61.5 KDa, respectively. Both proteins belong to the HMGR I category according to protein conserved domain prediction analysis. This HMGR category is located in the cytoplasm and is the first rate-limiting enzyme in the MVA pathway^6^. BLASTp search revealed that GbHMGR2 and GbHMGR3 proteins feature 84%–71% identities with the HMGR proteins of *G. biloba*, *Picea sitchensis*, *Taxus media*, *Trema orientalis*, *Ricinus communis*, and *Populus euphratica*.

**Figure 2:**
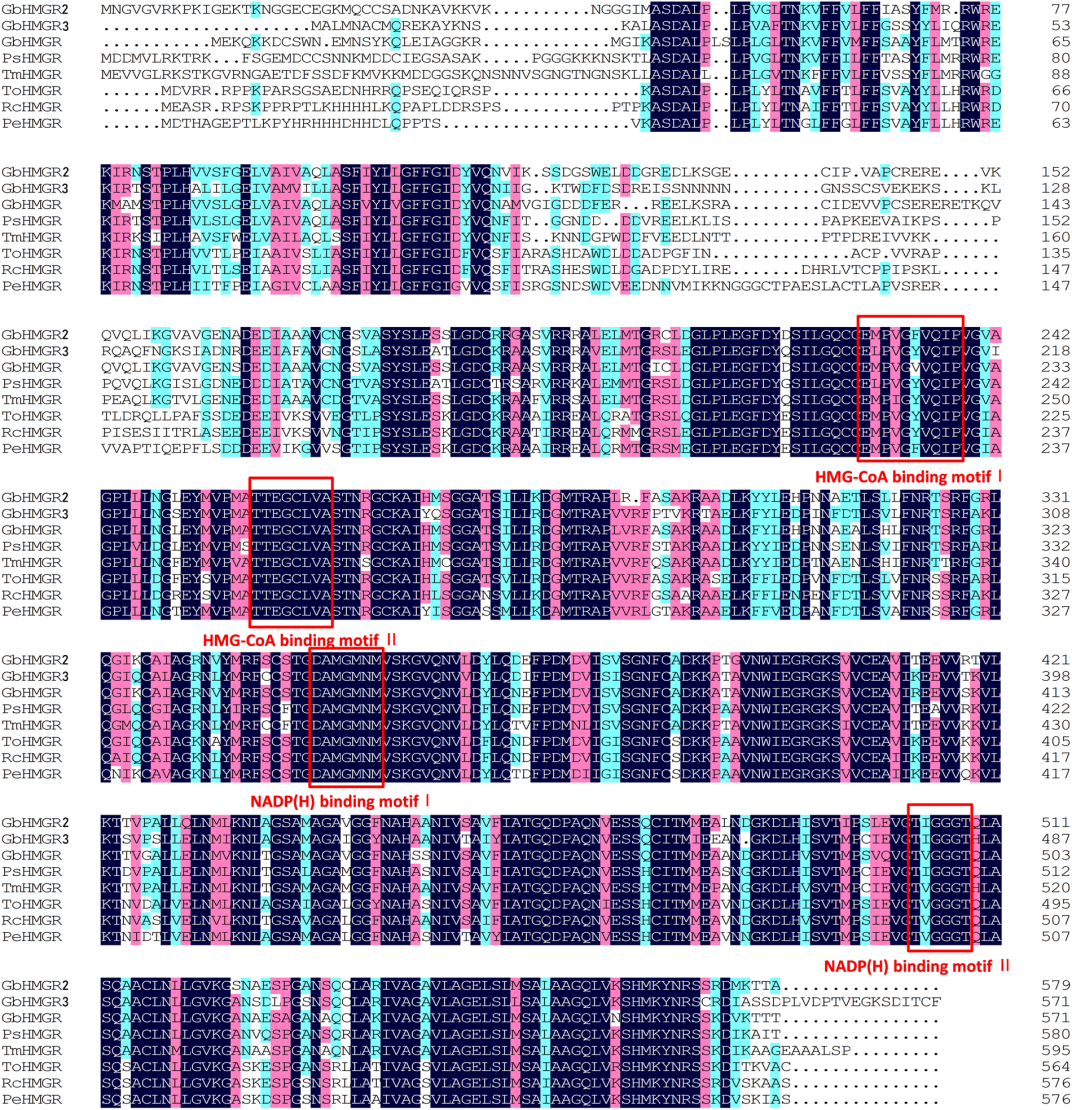
Multiple sequence alignments of GbHMGR2 and GbHMGR3 with other plant HMGR proteins. Dark blue: identity = 100%; red: 75% ≤ identity < 100%; light blue: 50% ≤ identity < 75%; Four conserved active sites of HMGRs, including the two NADP(H) binding motifs (TVGGGT and DAMGMNM) and two HMG-CoA binding motifs (TTEGCLVA and EMPVGYVQIP), are highlighted in red square box.

Transmembrane structural analysis of TMHMM Server 2.0 showed that the GbHMGR2 protein contains a transmembrane structure at positions 51–73 and 86–108, whereas those for GbHMGR3 protein are located at positions 26–48 and 61–83. Multiple alignment analysis revealed that GbHMGR2 and GbHMGR3 possess four conserved active motifs: two NADP(H) binding domains (TVGGGT and DAMGMNM) and two Hmg-CoA binding domains (TTEGCLVA and EMPVGYVQIP). These active motifs are highly conserved in the HMGRs from other plants and function as the catalytic active sites of the HMGR protein. Moreover, Glu of TTEGCLVA motif plays an important role in the HMGR catalytic action^18^ (Fig. 2). We speculate that GbHMGR2 and GbHMGR3 proteins are membrane-binding proteins with catalytic functions similar to those of other HMGR enzymes.

**Figure 3:**
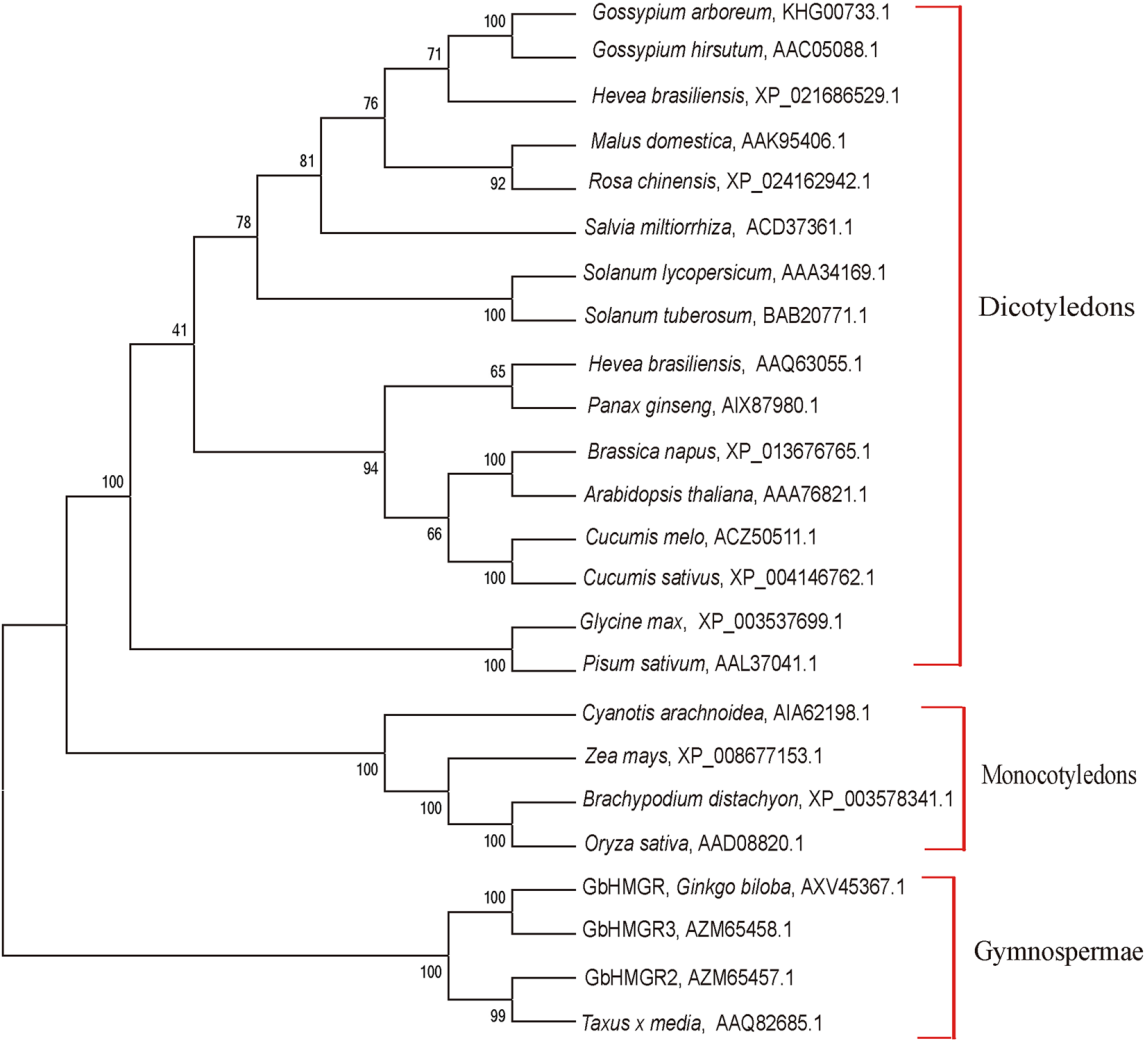
Phylogenetic tree of *HMGRs* from different species using the neighbor-joining method. The reliability of the tree is measured by bootstrap analysis with 1000 replications. The bars represent evolutionary distance.

A phylogenetic tree from 24 HMGR proteins with a common ancestor was constructed to further understand the evolutionary relationship between GbHMGR2, GbHMGR3, and other HMGR proteins. The tree was divided into three branches, namely, dicotyledon, monocotyledon, and gymnosperm (Fig. 3). GbHMGR, GbHMGR2, GbHMGR3, and HMGR from *T. media*, are clustered as gymnosperms. This result indicates the evolutionary diversity and conservatism of HMGR proteins.

### Promoter sequence analysis of *GbHMGR2* and *GbHMGR3*

The promoter sequences of *GbHMGR2* and *GbHMGR3* span lengths of 825 and 686 bp, respectively (Supplementary Figs. S1 and S2). Table 1 displayed the *cis*-acting elements of *GbHMGR2* and *GbHMGR3*. The TATA-box^19^ and CAAT-box^20^ were located at positions −28 bp and −173 bp of the *GbHMGR2* promoter region, respectively. In addition, four light-responsive elements, [i.e., I-Box (position −87 bp), Sp1 (position −677 bp), GT1-motif (position −357 bp), and chs-CMA 1a (position −730 bp)];^21–23^ a low-temperature-responsive (LTR) element (position −163 bp)^24^, a gibberellin-responsive element GARE-motif (position −314 bp)^25^, and a development-related element HD-Zip1 (position −717 bp)^26^ were detected in the *GbHMGR2* promoter region. This finding indicates that *GbHMGR2* is regulated by various external signals, such as light, cold, and hormones.

**Table 1:** Putative cis-elements in the promoters of *GbHMGR2* and *GbHMGR3*.

Element	Position	Signal Sequence	Expected function	Reference
***GbHMGR2***				
TATA-box	−28	TATAA	core promoter element around −30 of transcription start	^19^
I-box	−87	CGAGAAGGCG	part of a light responsive element	^21^
LTR	−163	CCGAAA	cis-acting element involved in low-temperature re	^24^
CAAT-box	−173	CAAT	common cis-acting element in promoter and enhancer regions	^20^
GARE-motif	−313	AAACAGA	gibberellin-responsive element	^25^
GT1-motif	−357	GGTTAA	light responsive element	^22^
Sp1	−678	CCTCCCTCTT	light responsive element	^21^
HD-Zip 1	−717	CAATAATTGCTCC	element involved in differentiation of the palisade mesophyll cells	^26^
chs-CMA1a	−730	TTACTTAA	part of a light responsive element	^23^
***GbHMGR3***				
TATA-box	−25	TATA	core promoter element around −30 of transcription start	^19^
AAGAA-motif	−59	GAAAGAA	cis-acting element involved in light responsiveness	^31^
MNF1	−152	GTGCCCTCCTGTTCGA	light responsive element	^23^
TGA-element	−206	AACGAC	auxin-responsive element	^30^
CAAT-box	−295	CAAACAACTCC	common cis-acting element in promoter and enhancer regions	^20^
ACE	−346	AAAACATTTA	cis-acting element involved in light responsiveness	^22^
Box III	−387	ATCATTTACACT	protein binding site	^31^
Box I	−421	TTTCAAA	light responsive element	^20^
ATCC-motif	−438	CAATCCTC	part of a conserved DNA module involved in light responsiveness	^21^
TGACG-motif	−592	TGACG	cis-acting regulatory element involved in the MeJA-responsiveness	^29^
TC-rich repeats	−656	ATTCTCTCCA	cis-acting element involved in defense and stress responsiveness	^27^
ERE	−682	ATTTCAAA	ethylene-responsive element	^28^

Similar to *GbHMGR2*, the TATA-box^19^ and CAAT-box^20^ were detected at positions −25 bp and −295bp of the *GbHMGR3* promoter region, respectively. Several other *cis*-acting elements, such as stress-responsive element TC-rich repeats (position −656 bp)^27^, light-responsive element ATCC-motif (position −438bp), Box I (position −421 bp), ACE (position −346 bp), MNF1 (position −152 bp)^21–23^, Eth-responsive element (ERE) (position −682 bp)^28^, MeJA-responsive element TGACG-motif (position −592 bp)^29^, auxin-induction-related element TGA-element (position −206 bp)^30^, conserved sequence AAGAA-motif (position −59 bp), and protein-binding site Box III (position −389 bp)^31^, were also predicted. Hence, the transcription of *GbHMGR3* was speculated to be regulated by light, cold, Eth, MJ, and auxin stress.

### Functional complementation of GbHMGR2 and GbHMGR3 in Saccharomyces cerevisiae

**Figure 4:**
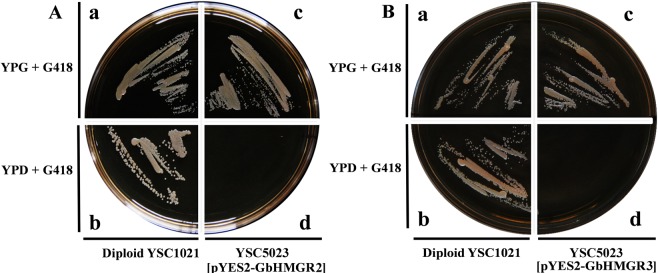
Functional complementation of GbHMGR2 and GbHMGR3 for the hmgr-deficient yeast YSC5023. (**A**) Functional complementation of GbHMGR2; (**B**) Functional complementation of GbHMGR3; (**a**) Diploid YSC1021 strain was grown on YPG + G418 medium; (**b**) Diploid YSC1021 strain was grown on YPD + G418 medium; (**c**) Haploid YSC5023 strain harboring pYES2-GbHMGR1 or pYES2-GbHMGR2 was grown on YPG + G418 medium; (**d**) Haploid YSC5023 strain harboring pYES2-GbHMGR1 or pYES2-GbHMGR2 failed to grow on YPD + G418 medium. The strains were grown at 28 °C for 2 days.

YSC5023 is an *hmgr*-deficient *S. cerevisiae* mutant strain that would die without mevalonate. We constructed yeast expression vectors, namely, GbHMGR2-pYES2 and GbHMGR3-pYES2 that could express GbHMGR2 and GbHMGR3, respectively, proteins through galactose induction to motivate MVA synthesis. Therefore, supplying MVA for YSC5023 can normalize the growth of the latter. As shown in Fig. 4, wild *S. cerevisiae* strain YSC1020 could grow on noninduced YPD (Fig. 4Ab,Bb) and induced YPG media (Fig. 4Aa,Ba), whereas the *S. cerevisiae* mutant *YSC5023* containing GbHMGR2-pYES2 or GbHMGR3-pYES2 could only grow on the induced YPG + G418 galactose medium (Fig. 4Ac,Bc). These results revealed that the functional deficiency of YSC5023 could be remedied by the expressions of *GbHMGR2* or *GbHMGR3*, indicating that GbHMGR2 and GbHMGR3 proteins can catalyze HMG-CoA to form MVA.

### TTL contents and expression profiles of *GbHMGR2* and *GbHMGR3* in the different organs of *G. biloba*

**Figure 5:**
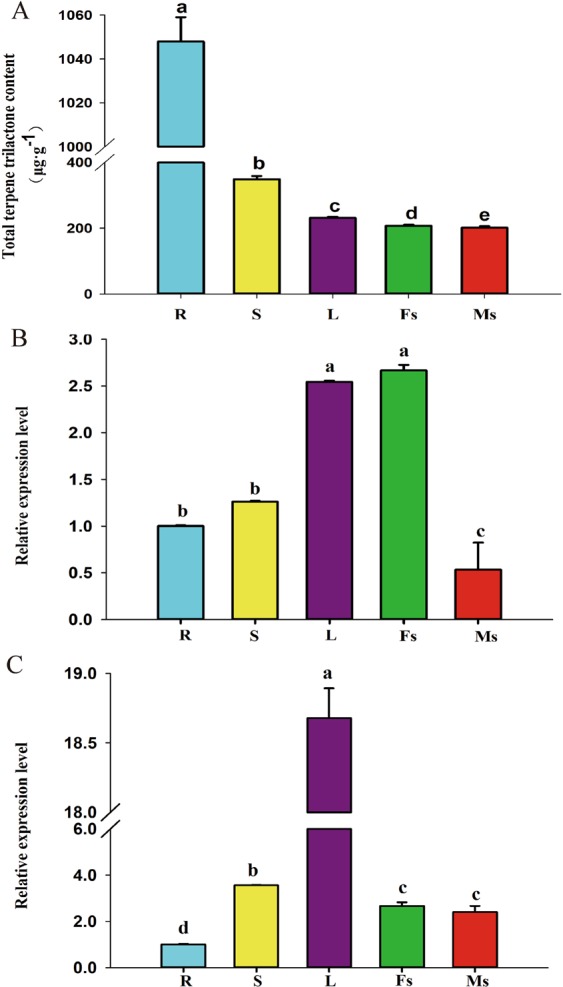
The accumulation pattern of TTLs and tissue expression patterns of *GbHMGR2* and *GbHMGR3* in different tissues of *G.biloba*. (**A**) Accumulation pattern of TTLs in different tissues; (**B**) Tissue expression patterns of *GbHMGR2*; (**C**) Tissue expression patterns of *GbHMGR3*. R: Root, S: Stem, L: Leaf, Fs: Female strobili, Ms: Male strobili; The gene expression level of *GbHMGR2* and *GbHMGR3* in the ginkgo root was set to 1. Data are shown as mean ± SE (n = 3); Means with different letters represent a Tukey’s honestly significant difference at *p* < 0.05.

The contents of TTLs remarkably varied in the different organs of *G. biloba*. As shown in Fig. 5A, the content of TTLs in the roots, which was fivefold of that in the male strobili, was the highest, followed by those in the stems and leaves. The lowest content was found in the male and female strobili, indicating that TTLs are mainly biosynthesized in the roots.

qRT-PCR results indicated that both *GbHMGR2* and *GbHMGR3* genes are differentially expressed in various organs. *GbHMGR2* was strongly expressed in the female strobili and leaves, followed by the stems and roots, and the lowest was observed in the male strobili (Fig. 5B). The expression pattern of *GbHMGR3* in the different organs of *G. biloba* considerably differed from that of *GbHMGR2*. As shown in Fig. 5C, the expression level of *GbHMGR3* in the leaves, which was 19-fold of that in the roots, was the highest, followed by those in stems, male and female strobili, and the lowest was noted in the roots.

### Changes in the TTL contents and expression levels of *GbHMGR2* and *GbHMGR3* in *G. biloba* leaves under dark, cold, MJ, SA, ABA, and Eth treatments

**Figure 6:**
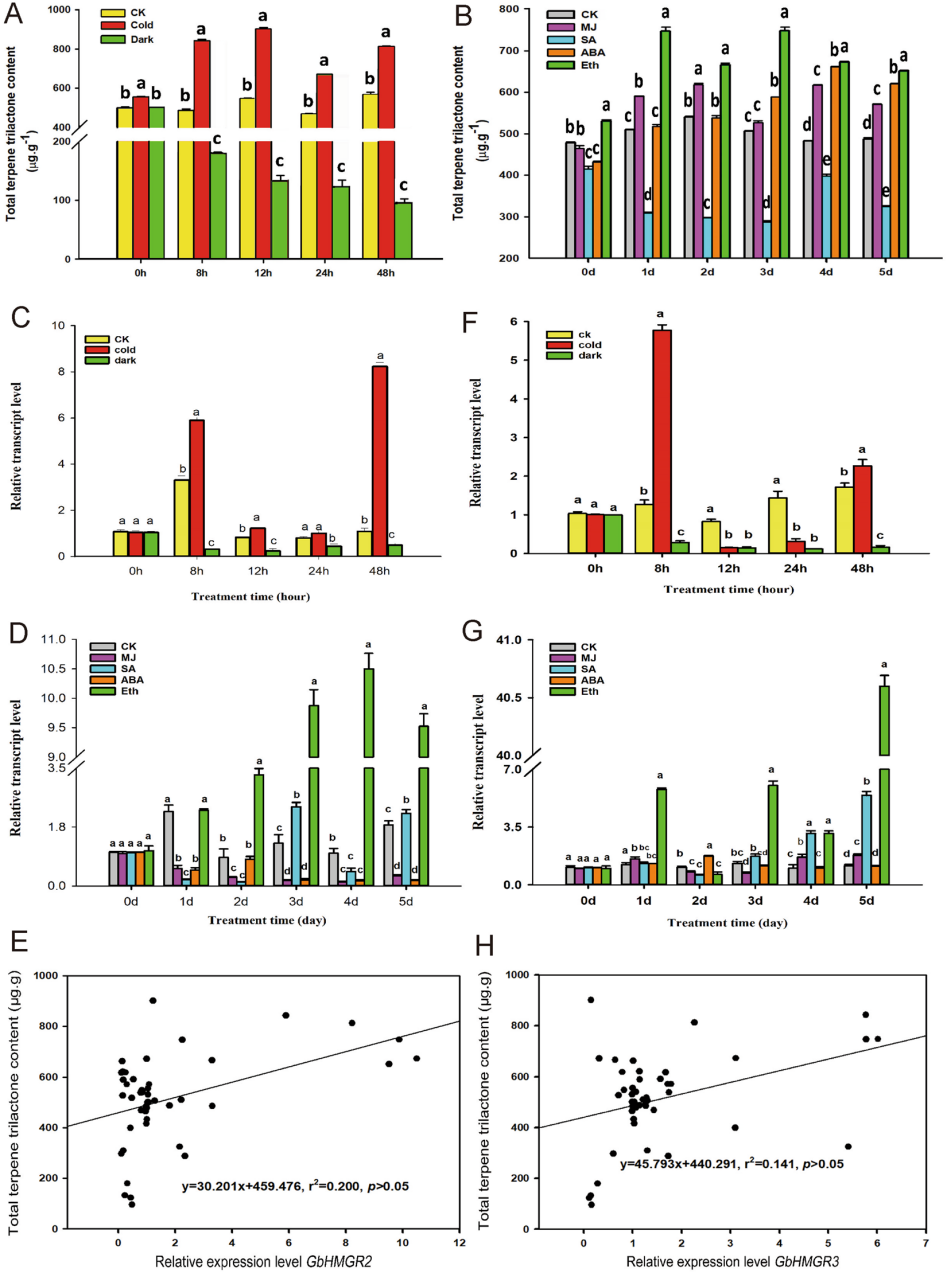
TTL content, expression changes of *GbHMGR2* and *GbHMGR3* under Dark, Cold, MJ, SA, ABA and Eth treatments, and correlation analysis of gene expression and TTL content. (**A**) TTL content changes by Dark and Cold; (**B**) TTL content changes by MJ, SA, ABA and Eth; (**C**) *GbHMGR2* expression level changes by Dark and Cold; (**D**) *GbHMGR2* expression level changes by MJ, SA, ABA and Eth; (**E**) correlation analysis of *GbHMGR2* expression and TTL content; (**F**) *GbHMGR3* expression level changes by Dark and Cold; (**G**) *GbHMGR3* expression level changes by MJ, SA, ABA and Eth; H: correlation analysis of *GbHMGR3* expression and TTL content. The expression levels were normalized to *GbGAPDH* gene. The transcript level of *GbHMGR2* and *GbHMGR3* at 0 h or 0 day post-treatment was set to 1. ALL the datas are shown as mean ± SE (n = 3); Means with different letters represent a Tukey’s honestly significant difference at *p* < 0.05.

As shown in Fig. 6A, the TTL contents were inhibited by dark treatment. The most substantial inhibitory effect was observed at 48 h after treatment at 16% of the control. Low temperature considerably increased the TTL content by 65% compared with that of the control at 12 h. Except SA, plant hormones enhanced the accumulation of TTL (Fig. 6B). The TTL content peaked to a value 1.28-fold of the control 2 days after MJ treatment and increased constantly after being treated with ABA and reached the maximum value, which is 27.8% higher than that of the control after 4 days. Eth treatment increased the TTL content to the maximum value, which is 47.7% higher than that of the control at 3 days after treatment. The TTL content decreased by 76.35% after 3 days of SA treatment, and the value obtained was lower than that of the control.

As shown in Fig. 6C, the *GbHMGR2* expression was substantially depressed under dark treatment and was 90% lower than that of the control at 8 h after treatment. Under cold treatment, its expression was enhanced by 6.6-fold compared with that of the control, and reached the highest level at 48 h. The expression level of *GbHMGR2* was considerably influenced by plant hormones (Fig. 6D), particularly, the induction by SA and Eth and inhibition by MJ and ABA. MJ treatment caused the continuous downregulation of *GbHMGR2* transcription level at only 5% that of the control at 4 days after treatment.Under SA treatment, the expression level of *GbHMGR2* initially decreased first and increased, and the value was 80% higher than that of the control at 3 days after treatment. The expression level of *GbHMGR2* was downregulated in response to ABA treatment, yielding a value 90% lower than that of the control at 5 days after treatment. Eth treatment induced the transcript of *GbHMGR2*, which continually increased and reached the highest expression value at 978% higher than that of the control at 4 days after treatment. Correlation analysis (Fig. 6E) showed that the changes in the expression of *GbHMGR2* and TTL content feature a positive but nonsignificant correlation.

As shown in Fig. 6F, the expression level of *GbHMGR3* was considerably downregulated under dark treatment, that is, 8% of the control at 24 h after treatment. However, this value was substantially enhanced under cold treatment. At 8 h after cold treatment, the transcription level of *GbHMGR3* sharply increased to a peak value that is 4.5-fold that of the control. All hormone treatments, namely, MJ, SA, ABA, and Eth, remarkably induced the *GbHMGR3* expression (Fig. 6G). *GbHMGR3* expression reached the maximum value, which is 1.67-fold that of the control at 4 days after treatment with MJ. ABA treatment showed similarly increasing values until a a moderate peak at 2 days after treatment. SA and Eth treatments distinctly upregulated the expression of *GbHMGR3* with the peak appering at 5 days after treatment. The former increased the expression by 3.74-fold and the latter by 34.6-fold compared with that of the control. Similar to *GbHMGR2*, a positive but nonsignificant correlation was observed between *GbHMGR3* and TTL content (Fig. 6H).

## Discussion

HMGR is one of the rate-limiting enzymes in MVA synthesis and catalyzes HMG-CoA to form MVA. This reaction can regulate terpene metabolism^32^. Plant *HMGR* genes typically contain multiple members, and the differences in the expression levels among various members can regulate the “carbon pool” in the MVA pathway^6,33^. In this study, two novel members of *Ginkgo HMGR* gene family, namely, *GbHMGR2* and *GbHMGR3*, were cloned and identified. The promoter sequence, tissue, and stress expression patterns of these two genes were analyzed. The catalytic functions of GbHMGR2 and GbHMGR3 were further verified by yeast functional complementation experiment. Screening the genes relevant to TTL biosynthesis and the increase in TTL content bears importance in genetic engineering.

The highest TTL was observed in roots, followed by those in the stems and leaves. This finding agrees with the result indicating that TTL is synthesized in roots, translocated to the shoots through the phloem, and then stored in leaves^27,28^. Previous studies have shown that different members of the *HMGR* gene family exhibit various expression patterns^33^. For example, in *Gentiana macrophylla*, *GmHMGR1* is expressed in the roots and flowers, whereas *GmHMGR2* is expressed in the leaves^34^. Meanwhile, the *PgHMGR1* and *PgHMGR2* genes of *Panax ginseng* are highly expressed in the seedlings and rhizophore but lowly expressed in the other tissues^35^. In the present study, we observed that *GbHMGR2* was highly expressed in the female strobili and leaves but lowly expressed in the male strobili. In addition, the expression level of *GbHMGR3* in the leaves was higher than that in the other tissues. Therefore, *GbHMGR2* and *GbHMGR3* exhibited different expression patterns. These findings were inconsistent with the pattern of the TTL content in the different tissues. Correlation analysis showed a positive but nonsignificant relationship between the TTL content and *GbHMGR2* or *GbHMGR3*. This results implied that *GbHMGR2* and *GbHMGR3* participated in the biosynthesis of TTLs in *Ginkgo*, but are not key genes. Diterpenes are the main components of TTLs in *Ginkgo*, and synthesized by the MEP pathway^5^.The biosynthesis of TTLs in *Ginkgo* is regulated by a complex network. The MVA and MEP pathways are the upstream pathways for the biosynthesis of TTLs, but genes in downstream pathway may play more important roles in this process. These may have caused the absence of significant correlations between GbHMGR2 or GbHMGR3 and TTL content. HMGRs have been reported to perform specific functions. For example, Song *et al*.^36^ proved that the overexpression of *HMGR* in *Lactococcus lactis* increases the production level of β-sesquiphellandrene by 1.25-fold to 1.60-fold. Kim *et al*.^37^ observed that the transformation of *Platycodon grandiflorum* with *Panax ginseng HMGR* (*PgHMGR*) induces a 1.2-fold to 1.5-fold increase in the levels of *HMGR* expression and total platycoside levels compared with those of the controls. Hence, we suggest that *GbHMGR2* and *GbHMGR3* might be involved in the biosynthesis of sesquiterpene and triterpene in *G. biloba*.

Gene expression is regulated to satisfy the requirements for the growth and development of plants and to resist external stress stimulated by the external environment stimulation. Darkness and low temperature are two usual abiotic stresses that affect the regulation of the plant *HMGR* genes. We previously showed that the *GbHMGR1* expression level and TTL content in the *Ginkgo* callus are higher under light than those under dark after 24 h of treatment, and the *GbHMGR1* expression and total TTL content were higher at 15 °C than at 24 °C^4^. These results are in accordance with that for *GbHMGR1*. Thus, *GbHMGR2* and *GbHMGR3* showed similar functions with *GbHMGR1*. Moreover, light- (i.e., I-box, Sp1, GT1-motif, and MNF1)^21–23^ and cold-responsive elements^24^ were found in the *GbHMGR2* promoter region; and light- (i.e., ATCC-motif, I-box, ACE, and MNF1) and stress-responsive elements (i.e., TC-rich repeats)^27^ were similarly found in the *GbHMGR3* promoter region. These findings explain the responses of *GbHMGR2* and *GbHMGR3* to cold and darkness.

Light corresponds with the synthesis of secondary metabolism in plants, but the effects vary among species. For example, in suspension-cultured *Lithospermum erythrorhizon* cells, white light strongly suppresses *HMGR* expression and shikonin formation^38^. Meanwhile, continuous dark exposure for 2–3 days increased the total ginsenoside content in a 3-year-old ginseng after the darkness-induced activity of *PgHMGR1*^7^. In the present experiment, the expression levels of *GbHMGR2* and *GbHMGR3* and the TTL content substantially increased in the *Ginkgo* plant exposed to light compared with those under darkness. This finding reflects the diversity among species. In a dark environment, plants are under carbon starvation^39^, and the leaves are induced to undergo accelerated senescence because of the breakdown of chlorophyll, decline in carbohydrate and protein contents, and ROS accumulation in the plant cells^40,41^. Therefore, dark stress might starve the *Ginkgo* seedlings, and the synthesis of TTLs would be limited by the decline in carbonaceous matters. From these results, we deduce that *GbHMGR2* and *GbHMGR3* could regulate the synthesis of TTL by responding to light in the *Ginkgo* leaves.

Low temperature can stimulate *Ginkgo* plants to accumulate several secondary metabolites to resist stress. Low temperature induces the expression of various genes, such as *GbPAL*^42^, *GbANS*^43^, and *GbFLS*^44^, which are involved in the synthesis of *G. biloba* flavonoids. Other plants, such as *Picrorhiza kurroa*, also exhibit a similar trend of upregulating *PkHMGR* and picroside content under low temperature^45^. The expression levels of *GbHMGR2* and *GbHMGR3* and the TTL content were significantly increased under low temperature, and this phenomenon corresponded with the cold motif in their promoter region. We observed that low temperature could enhance *GbHMGR2* and *GbHMGR3* transcript and stimulate the biosynthesis of TTLs to a certain extent.

Chemical elicitors, such as MJ, SA, ABA, and Eth, play key roles in the defense responses and development of plants. MJ is a well-known elicitor of secondary metabolism in plants^46^ and can elicit the accumulation of total phenols, flavonoids, and acacetin, a flavonoid compound with multiple pharmaceutical values^47^. Moreover, applying MJ induces the expression of the genes involved in terpenoid synthesis, such as *GbHMGS1*^48^, *GbHMGS2*^49^, and *GbIDS2*^50^. However, contrary to these genes, *GbHMGR2* expression was suppressed after treatment with MJ. This discrepancy suggests the tissue specificity of *GbHMGR2* expression, which presented a proportional expression level in the leaves and female strobili. Under MJ treatment, the TTL content in Ginkgo leaves substantially increased, and the expression of *GbHMGR3* was stimulated and was higher than that in the other tissues. This result was consistent with the MJ-responsive element TGAGG-motif in the *GbHMGR3* promoter region. This finding also implies that *GbHMGR3* is involved in the formation of TTLs in *Ginkgo* leaves. SA is another critical signaling molecule that is closely related to local and systemic acquired resistance (SAR)^51^. Numerous plant resistance genes, such as the genes we previously characterized (i.e., *GbANS*^43^, *GbFLS*^44^, and *GbHMGR*^4^), are upregulated in response to SA. The transcription levels of *GbHMGR2* and *GbHMGR3* increased 3 days after SA treatment. This finding indicates that *GbHMGR2* and *GbHMGR3* are involved in the SAR. The TTL content decreased after SA treatment, although the *GbHMGR2* and *GbHMGR3* transcriptions were induced. This phenomenon suggests that SA might inhibit the formation of other substances in the MEP and MVA pathways. These findings require further investigation.

ABA controls stomatal movement, seed dormancy, germination, and plant development^52^ and is closely related to *HMGR* gene expression^53^. For example, ABA signals modulate the expression and/or activity of *HMGR* to control the fruit growth and final fruit size of “Hass” avocado^54^. ABA also induces the production of several secondary metabolites. In ABA-treated *Artemisia annua* plants, artemisinin content was considerably increased, and the expression of important genes, such as *HMGR*, in the artemisinin biosynthetic pathway, was remarkably induced^55^. The results showed the inhibited *GbHMGR2* expression, slightly increased *GbHMGR3* expression in response to exogenous ABA, and considerably increased TTL content compared with that of the control. These asynchronous phenomena might be attributed to the multiple roles of ABA in plants. Yang *et al*.^56^ discovered that tanshinone production and the mRNA level of *HMGR* in *Salvia miltiorrhiza* were considerably enhanced by ABA treatments. Moreover, exogenous applications of ABA trigger endogenous MJ accumulation, whereas exogenous MJ can directly induce tanshinone production mainly via the MEP pathway in the hairy roots of *S. miltiorrhiza*. Similar to tanshinone, *Ginkgo* TTLs are synthesized through the MEP or MVA pathway. Therefore, we suggest that the ABA treatment might trigger endogenous MJ accumulation in *Ginkgo* leaves, thereby enhancing the TTL content. However, *GbHMGR2* and *GbHMGR3* expression showed no increase similar to those under the exogenous MJ treatment.

Eth is also an important hormone in the activation of plant defenses and forms a signal network with jasmonic acid, SA, and ABA^57^. *HMGR* expression is also modulated by Eth. Lv *et al*.^58^ reported that *MdHMGR2* from *Malus domestica* is significantly induced by Eth. Numerous studies indicated that Eth enhances the biosynthesis of terpenoids in plants. For example, Eth treatment increases ocimene, trans-caryophyllene, β-elemene, valencene, and α-panasinsene in citrus fruits^59^. In the present study, we noted that ERE was present in the promoter region of *GbHMGR3*. Eth treatment also drastically enhanced the expression levels of *GbHMGR2* and *GbHMGR3* and the TTL content. The results indicate that Eth induces the transcript of the novel genes and stimulates the biosynthesis of terpenoids in *Ginkgo* leaves.

## Materials and Methods

### Plant materials and treatments

Tissues from the roots, stems, leaves, and male and female strobili were collected from 30-year-old grafted *Ginkgo* trees (cv. Jiafoshou) from the Ginkgo Garden of Yangtze University, Jingzhou, China. The tissues were placed in a freezer at −80 °C after freezing in liquid nitrogen and used for RNA extraction.

Two-year-old grafted *Ginkgo* saplings were collected from the Ginkgo Garden of Yangtze University for the stress and hormone treatments, which included dark, cold (4 °C), MJ, SA, ABA, and Eth treatments. The saplings were placed in a completely dark plant growth chamber at 25 °C and 75% relative humidity (RH) for the dark treatment and in a luminous (600 μmol m^−2^·s^−1^) plant growth chamber at 4 °C and 75% RH with a 16 h/8 h light/dark photoperiod for the cold treatment. For the control, the *Ginkgo* saplings were exposed to light (600 μmol·m^−2^·s^−1^) with a 16 h/8 h light/dark photoperiod at 25 °C. The leaves of the treated *Ginkgo* saplings were sampled at 0, 8, 12, 24, and 48 h after treatment, frozen in liquid nitrogen, and placed in a freezer at −80 °C.

SA, ABA, MJ, and Eth were dissolved in 0.01% (v/v) Tween 20, and their final concentrations were 10, 100, 100, and 10 mM, respectively. The four hormone solutions were sprayed on both sides of the Ginkgo leaves for the hormone treatment group. For the control, 0.01% (v/v) Tween 20 was sprayed on the leaves. All the hormone-treated plants were placed in a growth chamber at 25 °C and 75% RH with a 16 h/8 h light/dark photoperiod. The leaves were harvested at 0–5 days after the hormone treatments, frozen with liquid nitrogen, and preserved in a freezer at −80 °C.

### Cloning of the full-length cDNA of *GbHMGR2* and *GbHMGR3*

The total RNA from each tissue was extracted using the TaKaRa MiniBEST Plant RNA Extraction Kit (TaKaRa, Dalian, China). The RNA extracted from the stems was used to synthesize the first-strand cDNA with the PrimeScript™ 1st Strand cDNA Synthesis Kit (TaKaRa, Dalian, China) according to the manufacturer’s instructions. Two pairs of specific primers, namely, GbHMGR2-UP and GbHMGR2-DN and GbHMGR3-UP and GbHMGR3-DN (Supplementary Table S1), were designed based on the unigene sequences of *HMGRs* from the *G. biloba* transcriptome database (GenBank accession number SRX1982952). The full-length cDNAs of *GbHMGR2* and *GbHMGR3* were amplified using single-strand cDNA as a template. *GbHMGR2* was amplified under the following conditions: 94 °C for 4 min, 32 cycles of 94 °C for 30 s, 54.7 °C for 30 s, and 72 °C for 90 s, and *GbHMGR3* was amplified under the following conditions: 94 °C for 4 min, 32 cycles of 94 °C for 30 s, 57.2 °C for 30 s, and 72 °C for 90 s. The PCR products were purified and ligated into the pMD19-T vector (Takara Bio Inc., Dalian, China). The recombined plasmids were transformed into *Escherichia coli* DH5α competent cells and sequenced by Shanghai Sangon Biotech.

### Promoter amplification of *GbHMGR2* and *GbHMGR3*

According to the ORF sequence of the *G. biloba GbHMGR2* and *GbHMGR3* genes, two round-nested pairs of primers, GbHMGR2-qd1 and GbHMGR2-qd2 and GbHMGR3-qd1 and GbHMGR3-qd2 (Supplementary Table S1), were designed near the 5′ end. The promoter sequences of *GbHMGR2* and *GbHMGR3* were obtained using the Universal Genome Walker Kit (Clontech, CA, USA), which mainly included adapter construction, DNA digestion, promoter walking library construction, and nested PCR.

### Bioinformatics analysis

A homologous search for the nucleic acid and predicted protein sequences of *GbHMGR2* and *GbHMGR3* was conducted with the online tools BLASTn and BLASTp in National Center for Biotechnology Information (https://blast.ncbi.nlm.nih.gov/Blast.cgi). The ORFs of *GbHMGR2* and *GbHMGR3* were predicted using Vector NTI 11.5. The molecular weights and isoelectric points of GbHMGR2 and GbHMGR3 were analyzed using an online tool, ExPASy (https://web.expasy.org/protparam/). The Conserved Domains Database (https://www.ncbi.nlm.nih.gov/Structure/cdd/wrpsb.cgi) and TMHMM Server V.2.0 (https://www.cbs.dtu.dk/services/TMHMM-2.0/) were used to analyze the protein conserved domain and transmembrane domain of GbHMGR2 and GbHMGR3. DNAMAN 8.0 software was used for the the translation of GbHMGR2 and GbHMGR3 proteins and multiple alignment of these proteins with the HMGRs other plants. The phylogenetic tree of HMGR was constructed using MEGA7.0. The transcription start site (TSS) and *cis*-acting element of the *GbHMGR2* and *GbHMGR3* promoters were predicted by Berkeley Drosophila Genome Project (http://www.fruitfly.org/seq_tools/promoter.html) and Plant Cis-Acting Regulatory Element (http://bioinformatics.psb.ugent.be/webtools/plantcare/html/).

### Functional complementation of *GbHMGR2* and *GbHMGR3* in *S. cerevisiae*

YSC5023 (*Δ[hmg2* + *hmg3]*) is an *hmgr*-double-deficient mutant that requires MVA and uracil to grow; this mutant is obtained by mating two *hmgr*-single-deficient mutants, BY4741 (hmg2) and BY4742 (hmg3), and dissecting the tetrad^60^. The functional deficiency of YSC5023 could be remedied by transferring exogenous *HMGR* genes into YSC5023, because *HMGR* expression can be induced by galactose and promote MVA synthesis. The growth state of recombinant YSC5023 yeast was observed to verify whether or not the proteins of GbHMGR2 and GbHMGR3 can catalyze HMG-CoA to form MVA. Two pairs of primers were designed according to the full-length *GbHMGR2* and *GbHMGR3* cDNA and the restriction enzyme cutting sites of the pYES2 plasmid. These primers were GbHMGR2-gnup and GbHMGR2-gndn and GbHMGR3-gnup and GbHMGR3-gndn (Supplementary Table S1), which contained the *Sca*I and *Xba*I restriction sites. The oding region was amplification through PCR, and the target fragments were purified with Agarose Gel DNA Purification Kit Ver. 4.0 (Takara, Dalian, China). The purified PCR products, which were double-digested by *Sca*I and *Xba*I, were ligated into the pYES2 vector. The recombinant plasmid was transformed into DH5α competence cells, and the positive strains were confirmed by sequencing in Shanghai Sangon (China). The constructed GbHMGR2-pYES2 and GbHMGR3-pYES2 expression vectors were transformed into YSC5023 using Frozen-EZ Yeast Transformation II Kit (Zymo Research, Orange, CA, USA). The recombinant yeasts were spotted on a yeast amino-acid-deficient medium, and the positively transformed strains were further confirmed by PCR. The wild-type *S. cerevisiae* strain YSC1020 and recombinant YSC5023 strain, which contained the GbHMGR2-pYES2 or GbHMGR3-pYES2 vector, were spread on noninduced YPD + G418 and induced YPG + G418 media and then incubated for 2 days at 28 °C. The growth of the strains was observed.

### Quantitative analysis of *GbHMGR2* and *GbHMGR3* gene expression

The expression levels of *GbHMGR2* and *GbHMGR3* were determined by qRT-PCR. RNA was extracted from the tissues and leaves of 2-year-old *Ginkgo* seedlings under different treatments by using TaKaRa MiniBEST Plant RNA Extraction Kit (TaKaRa, Dalian, China) and inverse transcribed using PrimeScript^TM^ RT Reagent Kit with gDNA Eraser (TaKaRa, Dalian, China). The primers (i.e., GbHMGR2-dlF and GbHMGR2-dlR and GbHMGR3-dlF and GbHMGR3-dlR) were designed with Primer Premier 5.0, and reference primers were GAPDH-F and GAPDH-R (Supplementary Table S1). LineGene 9600 Plus Fluorescent Quantitative PCR System and BioEasy Master Mix (SYBR Green) Kit (Bioer, Hangzhou, China) were used for qRT-PCR analysis. Three biological replicates were set for each treatment, and each sample was analyzed thrice. The relative expression levels of *GbHMGR2* and *GbHMGR3* were presented as 2^−ΔΔCt^ in line with the C_T_ method^61^. Data for qRT-PCR are shown as mean ± SE (*n* = 3).

### Determination of TTL content

The TTL content in the tissues and leaves of 2-year-old *Ginkgo* seedlings under different treatments were determined via high-performance liquid chromatography (UltiMate 3000, Thermo, USA) with evaporative light scattering detector (6000, Alltech, USA) according to the method of Ganzera *et al*.^62^.

### Statistical analysis

Data analysis was performed with one-way analysis of variance with Tukey’s honestly significant difference test by using SPSS 11.0 (SPSS Inc., USA) for Windows. The level of significance was set to *p* < 0.05.

## Supplementary information

Supplementary Figures and Table
