# Supplementary material for: Characterization and functional analysis of two novel 3-hydroxy-3-methylglutaryl-coenzyme A reductase genes (GbHMGR2 and GbHMGR3) from Ginkgo biloba
Source: Sci Rep. 2019 Oct 1;9:14109. doi: 10.1038/s41598-019-50629-8 (PMC6773693; doi:10.1038/s41598-019-50629-8)
Supplement: Supplementary file 1 — Supplementary Figures and Table [file 41598_2019_50629_MOESM1_ESM.pdf]

Characterization and functional analysis of two novel 3-hydroxy-3-methylglutaryl-coenzyme A reductase genes (*GbHMGR2* and *GbHMGR3*) from *Ginkgo biloba*

Shen Rao[1,2a], Xiangxiang Meng[1a], Yongling Liao[1,2a], Tian Yu[2,3], Jie Cao[1], Junping Tan[1], Feng Xu[1,2*], Shuiyuan Cheng[2*]

[1] College of Horticulture and Gardening, Yangtze University, Jingzhou 434025, China

[2] National R&D for Se-rich Agricultural Products Processing Technology, Wuhan Polytechnic University, Wuhan 430023, China

[3] Serun Health Industry Group, Enshi 445000, China

[a] The authors contributed equally to this study

***Corresponding authors**: Feng Xu, E-mail: xufeng198@126.com, Tel: +86 716 8066260, Fax: +86 716 8066262; Shuiyuan Cheng, E-mail: s_y_cheng@sina.com

```
-825    ACTATAGGGCACGCGTGGTCGACGGCCCGGGCTGGTAAAAATTATCACTATTTCATGGGA
-765    CTGTGACTATTTTTTGATGGCCCTACATTTACTTAACAATAATTGCTCCAAAAGAATAGG
                                    chs-CMA1a   HD-Zip 1
-705    AAGCTCCTCTGCATGGAACCTCCCTCTTATTGACATGGAAGGGATTGCGCTGATTGATTT
                                Sp1
-645    TTTATCAAATTTTTTACTTGTGGAACGCGGATTCAAACTCAAACCAAATGACTTAGGTTA
-585    TAACACATTAGAAATGTTAAAAGGCGAAACTTTCGCAACGATATAATTCGAAGGTGAACA
-525    ATGCTAGAAACTACTTACCTAACTATTAGGAACCCACTACACCCATTTGAGTGAATCTTA
-465    TAGGGTGGTCCCATTGAGGGGGCTAATTATGGAACATCATGGTCCCAAAAAATTGAAAAA
-405    GTGAAGCTCCTAATACTTAAAGGAGAAATTTTCGGAGAGAAGGGGTTAAATTTAGGCCTG
                                                  GT1-motif
-345    AACATTTGGAGAATCCATAGACACCTAAACAGAAATGATTTTTTTCTTTCTCTTCCATTT
                                    GARE-motif
-285    TTCACATTTGAACACTAAAAATTTGTGCCGCTATAATAACTATTCCTAAAATTATCACTG
-225    TTTCATAGGACCATGACTGTTTTTTTATGGCCCTACATTTACTTAGCAGCAATTGCTCCG
                                                        CAAT-box
-165    AAAGAATAGGAAACCCCAACCTCACTCTTATTGACATGGAAGGGATTGGGGCGAGGTACA
        LTR
-105    GGTGGAAGGCGAGAAGGCGATAGTGACCGTGTTCTTTGGGAATAAATTAATGAGCAATCA
                 I-box
-45     GGAGAGAGCCTCATATAATCTACTTTCCTTCCCATTCCACCGTCGAGAGGGCCCTTTGCT
                TATA-box                +1 Transcription Start
+16     TTGCATTCTATTGCATTGCATTGCATTGCATTGGAGCTTTCCAAACAAGTGTTTTGGAAG
+76     CGAGGTAGAATACCATGAATGGTGTTGGGGTGAGAAAACCAAAGATAGGGGAGAAGA
```

Supplementary **Fig. S1**. Promoter sequence of GbHMGR2. Cis-acting regulatory element, Transcription Start Site and initiation codon were marked with box.

```
-868    ACTATAGGGCACGCGTGGTCGACGGCCCGGGCTGGTATTTGATGGTTATATTTATTTATG
-808    AATCATAGCTTTATAAGATTATGATAGATTGATGACTTATAAAGCACAGATTAAAAAGTG
-748    CACTCAAACTAGAATGACTCATCAACATGCATATAAAACTTAAAACGATAAAGAAGTCTA
-688    TTTCAAAATCGAACCATGCCATGATTCTCTCCATTAATTGATAGATGTTGAAGCTCTCTC
        ERE                   TC-rich repeats
-628    TTGTTGAGCTACTAAGGATAAAATTAGTCTAATGACGACATGAAAGTTATATTGCTCCTA
                                        TGACG-motif
-568    CCACATTTGATATATGCACGAAGCCCTCATTGTTTTTATGAAATACATCTTGATATATAC
-508    ATTACCTTGTTAGGTCAGCATAATAAATTAAGGCAAGCAATCCACCAAATCAACACTTAC
-448    TCACAATCCTCACTCATGGATTTTCAAATTATCTTTATGGAAAACATCTCATCATTTACA
          ATCC-motif          Box I                          Box III
-388    CTTTGATCGAAAAGTGCTTAATGAATAATAAAAAAAACATTTAATGCTAGCCCCAATTAA
                                                ACE
-328    TGTGACTACATGGTGGAATATCACAAACAACTCCCTAGAATTAGGCCTTTTAGGAACTAA
                                CAAT-box
-268    TCTTGGTAAACAAGATAACAAACCCTCTTCGTAGCATTATGGTCCCCAAAAGCGGGAAAC
                                                             TGA-element
-208    GACCATATTCTCTTAGGCATCCAACTCTTCGGAACCGCGCTGTGCCCTCCTGTTCGAACA
                                                           MNF1
-148    CCTGTCACAAAGGTAACATTTGTTGTTTATTAGTTGGCAGAGTTTTATATTAGTAAGTGG
-88     TGTCTCTTATGTCTAGGCAGAGGGAAAGAACAGTACGATAGAGACGAGAGGCCTGACAAA
                               AAGAA-motif
-28     TATATGTAGAATAGATCAAGTTATAATCTTGGGCGTTTGCATGCAGTTCCTCTCTTTTTT
        TATA-box                    +1 Transcription Start
+33     TATTTCTATTTTTACTTGAATGCTATTCAGGCTAGATTTGTTTCTGTAATCCTGATCATC
+93     ATGGCATTGATGAATGCGTGTATGCAACGTGAGAA
```

Supplementary **Fig. S2.** Promoter sequence of GbHMGR3. Cis-acting regulatory element, Transcription Start Site and initiation codon were marked with box.

Supplementary **Table S1**. Primers used in this study.

Primer	Description	Sequence（5’→3’）
GbHMGR2-UP	GbHMGR2 forward primer for cDNA amplification	CATGAATGGTGTTGGGGTGA
GbHMGR2-DN	GbHMGR2 reverse primer for cDNA amplification	TCGGTCTCAGGCGGTTGT
GbHMGR3-UP	GbHMGR3 forward primer for cDNA amplification	CCTGATCATCATGGCATTG
GbHMGR3-DN	GbHMGR3everse primer for cDNA amplification	CCATCAGCCTAAGAAAAGCA
GbHMGR2-qd1	GbHMGR2 primer for promoter 1st round amplification	TCTTCTCCCCTATCTTTGGTTTTCTCA
GbHMGR2-qd2	GbHMGR2 primer for promoter 2nd round amplification	TCACATTCTCCACCATTCTTTGTCTTC
GbHMGR3-qd1	GbHMGR3 primer for promoter 1st round amplification	TTCTCACGTTGCATACACGCATTCATC
GbHMGR3-qd2	GbHMGR3 primer for promoter 2nd round amplification	CTTGTTGGTAAAGGCGACGGGCAGAG
AP1	Adapter primer，1st round	GTAATACGACTCACTATAGGGC
AP2	Adapter primer，2nd round	ACTATAGGGCACGCGTGGT
GbHMGR2-gnup	GbHMGR2 forward primer for yeast verification	CGAGTACTCATGAATGGTGTTGGGGTGA
GbHMGR2-gndn	GbHMGR2 reverse primer for yeast verification	CGTCTAGATCGGTCTCAGGCGGTTGT
GbHMGR3-gnup	GbHMGR3 forward primer for yeast verification	CGAGTACTCCTGATCATCATGGCATTG
GbHMGR3-gndn	GbHMGR3 reverse primer for yeast verification	CGTCTAGACCATCAGCCTAAGAAAAGCA
GbHMGR2-dlF	GbHMGR2 forward primer for qRT-PCR	GCAATGGGAGTGTGGCTTCTTA
GbHMGR2-dlR	GbHMGR2 reverse primer for qRT-PCR	ATCTGCCGGTCATCAACTCCA
GbHMGR3-dlF	GbHMGR3 forward primer for qRT-PCR	TGATACAGAGGTGGAGGGAGAAGA
GbHMGR3-dlR	GbHMGR3 reverse primer for qRT-PCR	TGAAGGAAGCAAGGAGAATGACC
GAPDH-F	Reference gene forward primer for qRT-PCR	GGTGCCAAAAAGGTGGTCAT
GAPDH-R	Reference gene reverse primer for qRT-PCR	CAACAACGAACATGGGAGCAT
M13F	Forward primer for bacterium solution PCR	TGTAAAACGACGGCCAGT
M13R	Rorward primer for bacterium solution PCR	CAGGAAACAGCTATGACC
